# *Pittosporum peridoticola* (Pittosporaceae), a new ultramafic obligate species restricted to Kinabalu Park (Sabah, Malaysia)

**DOI:** 10.1186/s40529-016-0119-9

**Published:** 2016-02-01

**Authors:** John B. Sugau, Antony van der Ent

**Affiliations:** 1 Sabah Forestry Department, Forest Research Centre, Jalan Sepilok, Sepilok, 90175 Sandakan, Sabah Malaysia; 2 Laboratoire Sols et Environnement, Université de Lorraine-INRA, UMR 1120, Nancy, France; 3 Centre for Mined Land Rehabilitation, Sustainable Minerals Institute, The University of Queensland, Brisbane, QLD 4072 Australia

**Keywords:** Hyper-endemic, Mount Tambuyukon, Soil chemistry, Ultramafic obligate

## Abstract

**Background:**

Kinabalu Park, in Sabah (Malaysia) on Borneo Island, is renowned for the exceptionally high plant diversity it protects, with at least 5000 plant species enumerated to date. Discoveries of plant novelties continue to be made in Sabah, especially on isolated ultramafic outcrops, including in the genus *Pittosporum* (Pittosporaceae) with *P. linearifolium* from Bukit Hampuan on the southern border of the Park, and *P. silamense* from Bukit Silam in Eastern Sabah, both narrow endemics restricted to ultramafic soils.

**Results:**

A distinctive new species of *Pittosporum* (*P. peridoticola* J.B.Sugau and Ent, *sp. nov.*) was discovered on Mount Tambuyukon in the north of Kinabalu Park during ecological fieldwork. The diagnostic morphological characters of this taxon are discussed and information about the habitat in which it grows is provided. The soil chemistry in the rooting zone of *P. peridoticola* has high magnesium to calcium quotients, high extractable nickel and manganese concentrations, but low potassium and phosphorus concentrations, as is typical for ultramafic soils. Analysis of foliar samples of various *Pittosporum*-species originating from ultramafic and non-ultramafic soils showed a comparable foliar elemental stoichiometry that is suggestive of ‘Excluder-type’ ecophysiology.

**Conclusion:**

*Pittosporum peridoticola* is an ultramafic obligate species restricted to Kinabalu Park with only two known populations within the boundaries of the protected area. It is vulnerable to any future stochastic landscape disturbance events, such as forest fires or severe droughts, and therefore its conservation status is ‘Near Threatened’.

## Background

Kinabalu Park, in the northwestern part of the Malaysian state of Sabah (on Borneo Island), is renowned for the exceptionally high plant diversity it harbours, with over 5000 plant species enumerated from an area less than 1200 km^2^ (Beaman 2005; Van der Ent et al. 2015a). Ultramafic outcrops (also called ‘serpentine’ or ‘ultrabasic’) are known globally for hosting distinctive and highly endemic floras (Brooks 1987). Ultramafic soils are characterised by unusual chemical characteristics, including high concentrations of potentially phytotoxic elements (nickel, cobalt, chromium), low concentrations of essential plant nutrients, and cation imbalances (high magnesium to calcium quotients) (Proctor 2003).

Ultramafic outcrops are widespread in Sabah, and occur in Kinabalu Park, predominantly on Mount Tambuyukon, and on the southern slope of Mount Kinabalu (Van der Ent et al. 2015a). In Kinabalu Park, ultramafic soils are known for high levels of species-richness and (local) endemism (Beaman and Beaman 1990). Overall, the vegetation on ultramafic soils often has a relative shorter stature and more open aspect, compared to vegetation on non-ultramafic soils. On tropical isolated mountains the altitudinal vegetation zonation is often compressed (Proctor et al. 1988; Bruijnzeel et al. 1993). This effect is particularly apparent on Mount Tambuyukon (2579 m asl), where the vegetation on the summit ridge is a graminoid dwarf scrub. Such dwarf scrub also occurs locally on the Mount Kinabalu massif, such as at Marai Parai, Bukit Babi and the Mentaki Ridge (Van der Ent et al. 2015a). At similar elevations on soils derived from granite or sandstone geology, relatively tall forest occurs. As part of ecological fieldwork undertaken in 2010–2013 a distinctive *Pittosporum* was found on the main summit ridge of Mount Tambuyukon, which is described here.

## Methods

In this article we use the Biological Species Concept (BSC), which is based on considering discontinuities in morphological variation as an indirect assessment of reproductive isolation and lack of gene flow preventing lineages from homogenising (Coyne and Orr 2004; Mallet 2008). Dried plant specimens were examined from the Sabah Parks Herbarium (SNP). Soil samples (n = 9) were collected near individuals of *P. peridoticola* on the summit ridge of Mount Tambuyukon, air-dried at room temperature and digested with concentrated nitric and hydrochloric acid in a specialized microwave (Milestone Start D). Foliar samples of *P. ferrugineum* (n = 1), *P. linearifolium* (n = 3), *P. longisepalum* (n = 1), *P. resiniferum* (n = 1), *P. peridoticola* (n = 1) were collected in Kinabalu Park and the nearby Bukit Hampuan Forest Reserve, air-dried at room temperature and similarly digested with concentrated nitric acid and hydrogen peroxide in a specialized microwave. Both soil and leaf sample digest solutions were then measured with an ICP-AES (Varian Vista Pro II) for nickel, cobalt, manganese, iron, magnesium, calcium, sodium, potassium and phosphorus. The ICP-AES instrument was calibrated using a 6-point multi-element standard prepared in each solution.

## Results

### Distribution and ecology of the genus *Pittosporum* in Sabah

The family Pittosporaceae consists of nine genera, of which the genus *Pittosporum* is the sole representative in Sabah (Malaysia, on the island of Borneo). The genus (approximately 200 species) ranges from tropical areas in Africa, South and East Asia and Australasia to the Pacific Region. All *Pittosporum* taxa are trees and woody shrubs growing to 2–30 m tall. In Sabah a total of six species have been described to date (Sugau 1994; 1995): *Pittosporum ferrugineum* Aiton, *P. linearifolium* Sugau, *P. longisepalum* Bakker, *P. ramiflorum* Zollinger ex. Miquel, *P. resiniferum* Hemsley and *P. silamense* Sugau. Of these *P. ferrugineum* is relatively widespread in Sabah, whereas *P. ramiflorum, P. resiniferum* and *P. longisepalum* are principally known from Kinabalu Park in Sabah, but also occur elsewhere in Southeast Asia. Two species, *P. linearifolium* and *P. silamense,* are hyper-endemics restricted to isolated ultramafic outcrops in Sabah on Bukit Hampuan and Bukit Silam respectively (Sugau 1994).

### Species description

**Fig. 1:**
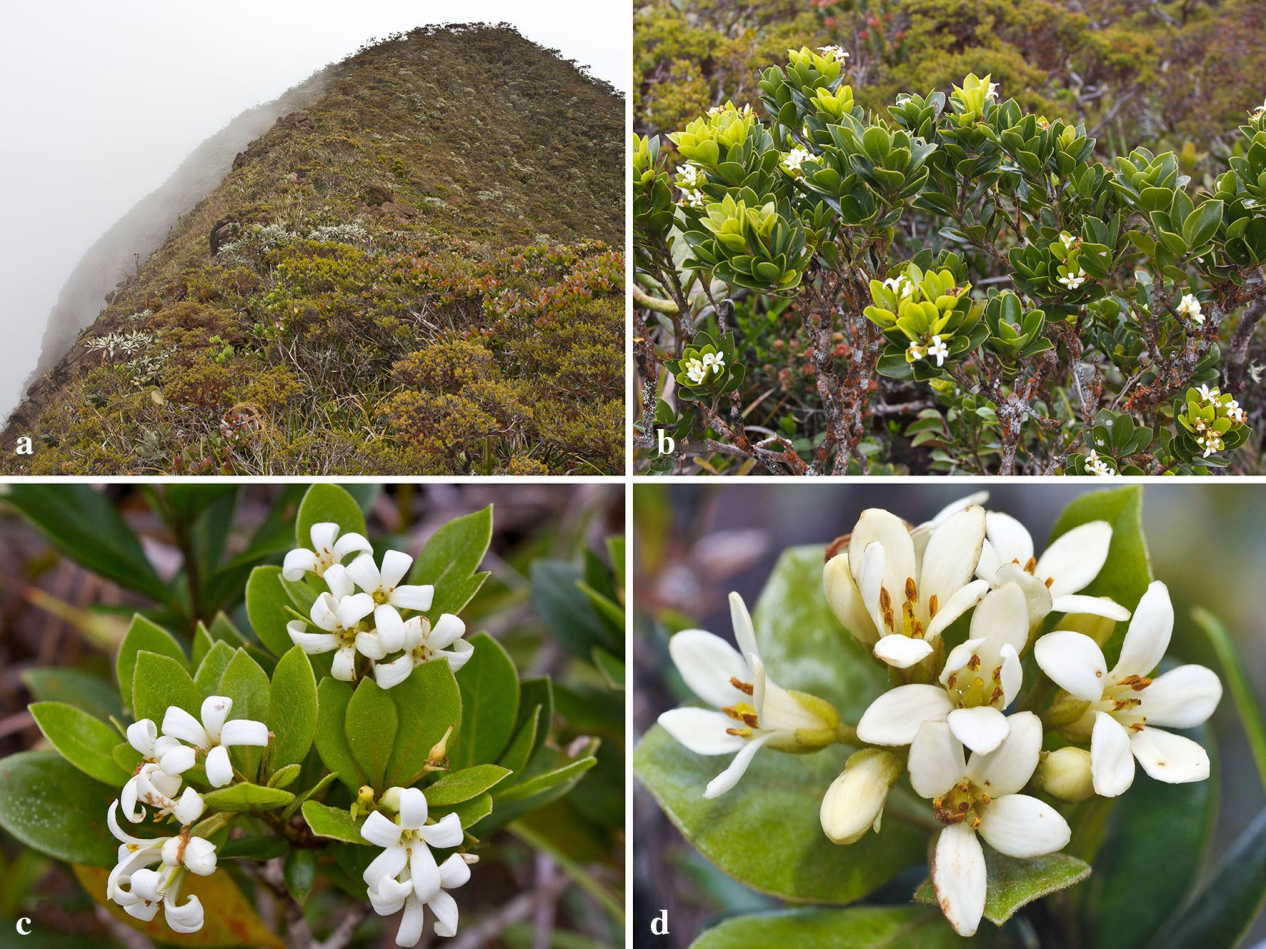
*Pittosporum peridoticola* in the field: (**a**) Habit of plant growing on ultramafic bedrock; (**b**) Whole plant; (**c**) Inflorescence; (**d**) Detail of inflorescence. (Photos by A. van der Ent and R. van Vugt)

**Fig. 2:**
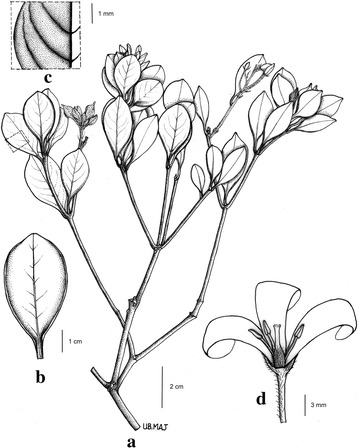
(**a**) The leafy twig with flower buds; (**b**) Leaf showing the slightly recurved margin; (**c**) Close-up of upper leaf surface showing finely impressed secondary veins. (A–C, SNP 26812) (**d**) Flower, showing the floral parts including the hairy ovary (two sepals, two petals, and two stamens removed) (SNP 25748)

*Pittosporum peridoticola* J.B.Sugau and Ent, *sp. nov.* (Figures 1 and 2).

The new species is morphologically similar to *P. longisepalum* Bakker in having finely impressed veins on the upper leaf surface and slightly recurved margin when dry but differs by its smaller obovate leaves, absent or very short (to 3 mm long) grooved petioles, shorter sepals (less than 5 mm long), longer staminal filaments (5 mm long), style (c. 2.5 mm) and petals (6–16 mm long). *Pittosporum longisepalum* has larger (4–6 long, 1.5–2 cm wide) elliptic leaves, longer (up to 5 mm long) sub-terete petioles, longer sepals (c. 7 mm long), shorter staminal filaments (c. 3 mm long), style (c. 1.5 mm long) and petals (c. 8 mm long). A comparison of taxonomical characters between *P. longisepalum* and *P. peridoticola* is provided in Table 1.

**Table 1:** Comparison of taxonomical characters between *Pittosporum longisepalum* and *P. peridoticola*

Character	*P. longisepalum*	*P. peridoticola*
*Leaf size (cm)*	4–6 × 1.5–2	2–4 × 0.8–2
*Leaf shape*	Elliptic	Obovate
*Leaf apex*	Acute to acuminate	Rounded, acute to shortly cuspidate
*Leaf base*	Cuneate, decurrent	Cuneate, decurrent
*Leaf hairiness*	Slightly pubescent beneath	Glabrescent, glabrous when mature
*Veins in upper surface*	Secondary veins slightly impressed	Secondary veins slightly impressed
*Number of veins*	5–7 pairs	4–6 pairs
*Flowering habit*	Among leaves	Among leaves
*Petal length (mm)*	~8	6–16 mm
*Ovary*	Hairy	Hairy
*Sepal length (mm)*	~7	3–4 mm
*Peduncle length (cm)* *+* *hairiness*	2–3; hairy	1–2.5; hairy
*Leaf margin*	Entire; recurved	Entire; slightly recurved
*Petiole (mm)*	~5	Very short to c. 3
*Filament length (mm)*	~3	5 mm
*Anther*	Oblong; apiculate	Oblong; apiculate
*Style*	~1.5 mm	2.5 mm
*Stigma*	Capitate	Capitate

Type—*A. van der Ent* et al. SNP 25748, Malaysia. Sabah. Kinabalu Park, Mount Tambuyukon, 14 April 2011 (holotype SNP; isotype SAN).

Shrub, 1.5–2 m tall, multi-branched, stem barely 4–5 cm thick at base. Young parts hairy. Leaves spirally arranged, borne on distal 15–20 cm of the branches, light green, obovate, 2–4.5 × 0.8–2 cm, coriaceous, brown to dark brown on both sides when dry, hairy (when young), glabrous when mature; apex acute to shortly cuspidate, base cuneate and typically decurrent (to the base of petiole), margin entire, slightly recurved when dry, midrib sunken on upper surface of lamina, raised on lower surface, secondary veins 4–6 pairs, not very conspicuous, finely impressed on upper leaf surface. Petiole absent or very short, to c. 3 mm long, grooved. Inflorescence terminal, a simple thyrse, with up to eight flowers. Peduncle 1-2.5 cm long, hairy, pedicel hairy. Flowers with sepals, petals and stamens 5-merous; sepals lanceolate, 3–4 mm long, hairy; petals oblong, 6–16 × 2 mm, white; stamens c. 6 mm long, filaments 5 mm long, anthers oblong, c. 1.5 mm long, apiculate; ovary ellipsoid, c. 3 mm long, densely hairy; style c. 2.5 mm; stigma capitate. Fruit not seen.

### Additional specimen examined

*Malaysia. Borneo, Sabah. Kinabalu Park*. SNP 27094—Mentaki Ridge; SNP 26812 and SNP 26781—Mount Tambuyukon, Musang Camp to Summit ridge; SNP 2574—Tambuyukon ridge second peak; SNP 30597. Specimens were all flowering and collected by *Van der Ent* et al.

### Distribution and habitat

*Pittosporum peridoticola* is found exclusively in ligneous scrub on extreme ultramafic soil on Mount Tambuyukon and Mount Kinabalu. The sites where it occurs are located at 1700–2500 m asl in dense vegetation consisting of shrubs (1–2 m tall). Co-occurring species include: *Styphelia malayanus* Jack subsp. *malayanus* (Ericaceae), *Phyllocladus hypophyllus* Hook. f. (Phyllocladaceae), *Weinmannia clemensiae* Steenis (Cunoniaceae)*, Leptospermum javanicum* Blume (Myrtaceae), *Wikstroemia indica* (L.) C.A.Mey. (Thymelaeaceae), *Lithocarpus rigidus* Soepadmo (Fagaceae), *Podocarpus brevifolius* (Stapf) Foxw. and *Dacrydium gibbsiae* Stapf (Podocarpaceae). On the summit ridge of Mount Tambuyukon, *Pittosporum peridoticola* was found in an area of just a few hundred m^2^ and only a small number of individuals were seen. Similarly, the habitat on Mount Kinabalu (Mentaki Ridge) is very small, comprising of only a few individuals. Other rare regional species are also known only from populations on ultramafic soils in Kinabalu Park, for example *Drosera ultramafica* A.Fleischm., A.S.Rob. and S.McPherson (Droseraceae), *Nepenthes rajah* Hook.f. (Nepenthaceae), *Calanthe otuhanica* C.L.Chan and T.J.Barkman (Orchidaceae) and *Weinmannia clemensiae* Steenis (Cunoniaceae).

### Etymology

The specific epithet “*peridoticola*” denotes the peridotite (ultramafic) bedrock on which this species grows on Mount Tambuyukon and Mount Kinabalu (from ‘peridotite’ the ultramafic bedrock, and; cola (Latin)—to dwell or inhabit). Peridotite is a dense, coarse-grained igneous rock, consisting of olivine and pyroxene minerals (magnesium-iron-silicates). This rock-type, to varying degrees serpentinised, is the main bedrock of ultramafic outcrops in Sabah, and forms the Mount Tambuyukon massif.

Key to *Pittosporum* species in Sabah and Sarawak (vegetative characters):

*1a.* Leaves very narrowly elliptic to linear, mostly 0.5–1.1 cm wide *P. linearifolium.*

*1b.* Leaves elliptic to obovate, typically wider (more than 1.5 cm wide ………….2.

*2a.* Leaves persistently densely hairy on the lower side, and conspicuously bullate between the veins on the upper side; margins markedly recurved when dry ………….*P. silamense.*

*2b.* Leaves never persistently hairy (glabrous or, if hairy, only when very young), and not bullate or veins only finely impressed on the upper side; margins not recurved or slightly recurved when dry ………….3.

*3a.* Veins on the upper leaf surface finely impressed; margin slightly recurved ………….*4*.

*3b.* Veins on the upper leaf surface flat, not impressed; margin not recurved ………….*5*.

*4a.* Leaves elliptic; petiole c. 5 mm long, sub-terete; sepals 6–7 mm long in flower ………….*P. longisepalum.*

*4b.* Leaves obovate; petiole very short to c. 3 mm long, grooved; sepals less than 5 mm ………….*P. peridoticola.*

*5a.* Leaves elliptic, or if oblanceolate to obovate then the apex gradually acuminate. Inflorescences borne in the leafy portions of branches ………….*P. ferrugineum.*

*5b.* Leaves markedly obovate, the apex abruptly cuspidate. Inflorescences borne on bare branches ………….6.

*6a.* Petioles 8–18 mm long. Flower larger, the petals 9–12 mm long. Infructescence peduncle not conspicuous or up to 0.5 cm long only. Mature fruits 1.7–2 cm long ………….*P. resiniferum.*

*6b.* Petioles longer, 15–25 mm long. Flower smaller, the petals only 4–7 mm long. Infructescence peduncle longer, 1–3 cm long. Mature fruits 1–1.5 cm long ………….*P. ramiflorum.*

### Soil and foliar chemistry

The analysis of a soil sample collected between the roots of *P. peridoticola*, and foliar analysis of different *Pittosporum* spp. are presented in Table 2. The results show that the soil chemistry from the rooting zone of *P. peridoticola* is extreme in containing very high magnesium (Mg), nickel (Ni) and manganese (Mn), but relatively low calcium (Ca) and potassium (K) concentrations. This chemistry is characteristic for ultramafic soils, and the soils on which it occurs are ‘hypermagnesian soils’ (*Hypermagnesic Cambisols*) (Van der Ent and Wood 2012). These soils occur at high elevation (1700–2500 m asl.), are very shallow and skeletal and derive from primary weathering of the bedrock close to the surface. They have high Mg:Ca quotients, low CEC , high extractable Ni and Mn concentrations , and are moderately acidic. Total soil K and P concentrations are low 52 and 118 μg g^−1^ respectively. As such these soil properties might induce phytotoxicity and nutrient deficiencies, and *P. peridoticola* must have evolved adaptations to these adverse soil chemical conditions.

**Table 2:** Foliar and soil chemistry of *Pittosporum* species (mean values in μg g^−1^)

Species	Al	Ca	Co	Cr	Fe	K	Mg	Mn	Na	Ni	P
*P. ferrugineum* (n = 1)	38	12,145	1.8	2.5	22	2080	1650	1080	10,330	3.3	275
*P. linearifolium* (n = 3)	34	8860	5	4	43	4690	4420	334	3790	8	484
*P. longisepalum* (n = 1)	26	18,730	1.1	1.6	31	8825	2540	1095	6600	10	443
*P. resiniferum* (n = 1)	34	29,080	0.7	1.8	18	4900	8710	381	67	3.4	422
*P. peridoticola* (n = 1)	27	12,480	16	0.7	18	6955	7200	195	1329	13	335
*Soil P. peridoticola (n* *=* *9)*	*4800*	*674*	*284*	*1293*	*169,500*	*52*	*25,420*	*5070*	*55*	*1571*	*118*

The soil is from the habitat of *Pittosporum peridoticola* on Mount Tambuyukon

The foliar analyses show that *Pittosporum* spp. are effective at excluding potentially phytotoxic elements (Ni, Mn, Mg) from the shoot and at the same time are efficient in sequestering essential elements (Ca, K, Na, P). Foliar accumulation of most elements is rather similar between the *Pittosporum* spp. despite the fact they grew in different habitats and on different soils. The leaf samples from *P. ferrugineum, P. longisepalum* and *P. resiniferum* originate from non-ultramafic soils, and these soils generally have higher concentrations of Ca, K and P compared to ultramafic soils. Nevertheless, the concentrations of foliar elements are either similar or erratic compared to *Pittosporum* spp. from ultramafic soils, and this points towards ‘Excluder-type’ ecophysiology (*sensu* Baker 1981) of this genus on a wide range of soil types.

## Discussion and conclusion

### Ecological and evolutionary aspects

Ultramafic outcrops may be conceptualized as ‘edaphic islands’ (Kruckeberg 1991; Rajakaruna 2004), and as such these insular features in the landscape can confer genetic isolation, which in association with locally strong climactic and edaphic stresses, may promote plant speciation and endemism (Kruckeberg 1986; Wong 2011; Rajakaruna and Boyd 2008). The combination of isolated ultramafic soils in combination with (high) elevation has been shown to be particularly conductive for the evolution of local endemics (‘neo-endemics’) on Mount Kinabalu, for example in the genera *Dendrochilum* (Orchidaceae), *Elatostema* (Urticaceae), *Rhododendron* (Ericaceae), *Leptospermum* (Myrtaceae), and *Nepenthes* (Nepenthaceae) (Barkman and Simpson 2001; Argent et al. 2007; Beaman 2001; Lee and Lowry 1980; Van der Ent et al. 2015b). Neo-endemic species are thought to have evolved in situ from related precursor taxa, and often form high-elevation and lower-elevation species-pairs. Other species are paleo-endemics, that were formerly widespread, but continue to persist locally when competition pressure is low (Stebbins and Major 1965; Anacker 2014).

The new species *P. peridoticola* is the third narrowly endemic species in the genus in Sabah that is obligate (restricted) to ultramafic soils (together with *P. linearifolium* and *P. silamense*). All three species are each known from isolated mountain summits (Mount Tambuyukon/Mentaki Ridge, Bukit Hampuan and Bukit Silam respectively). The habitat of all three species is very open forest or dwarf scrub. This suggests a high light requirement permitted through competitive exclusion due to habitat specialisation under strong edaphic stresses. *Pittosporum peridoticola* in particular appears to have a slow growth rate, judging from the thick woody gnarled stems, and co-occurs with a number of other plant species endemic to Mount Tambuyukon, such as *Eriobotrya balgooyi* K.M.Wong and Ent (Rosaceae) (Wong and Van der Ent 2014), *Gynura tambuyukonensis* Vanij. and Ent (Asteraceae) (Van der Ent and Vanijajiva (Van der Ent and Vanijajiva 2014), *Rhododendron meijeri* Argent, A.L.Lamb and Phillipps and *R. baconii* Argent, A.L.Lamb and Phillipps (Ericaceae) (Argent et al. 2007). Other hyper-endemic species from Mount Tambuyukon include the recently described *Begonia moneta* C.-I Peng, Rimi and C. W. Lin and *B. peridoticola* Rimi, C.-I Peng and C. W. Lin (Begoniaceae) (Peng et al. 2015). The situation of ultramafic obligate *Pittosporum*-species in Sabah mirror that of an another genus of woody shrubs, *Timonius* (Rubiaceae), of which four species are currently understood as ultramafic obligates: *T. tambuyukonensis* J.Chen, *T. stenolobus* J.Chen and K.M.Wong, *T. leopoldii* J.Chen and K.M.Wong and *T. ophioliticus* J.Chen, with the first three species probably endemic to Kinabalu Park (Chen et al. 2014). Future molecular studies might elucidate the phylogenetic position of the ultramafic obligate *Pittosporum*-species in Sabah.

### Conservation status

The habitat of *P. peridoticola* lies entirely in Kinabalu Park (an IUCN Category II National Park) and it is therefore statutorily protected. Nevertheless the restriction of this species to just two known populations on Mount Tambuyukon and Mount Kinabalu respectively, and the inherent small total number of individuals means that this species is vulnerable to any future stochastic events, such as forest fires or severe droughts that could ultimately lead to the extinction of this rare taxon. However, as there is no currently available evidence of decline or fluctuations in the extent of its occurrence (EOO), the area of occurrence (AOO), or the number of mature individuals, the conservation status of the species is currently classified as ‘Near Threatened’ (IUCN 2001).
